# The sociocultural framing of public attitudes to sharing the costs of social care for older people in England

**DOI:** 10.1111/hsc.13946

**Published:** 2022-08-05

**Authors:** Josie Dixon, Josephine Exley, Gerald Wistow, Raphael Wittenberg, Martin Knapp, Nicholas Mays

**Affiliations:** ^1^ Care Policy and Evaluation Centre (CPEC), Department of Health Policy London School of Economics and Political Science London UK; ^2^ Policy Innovation and Evaluation Research Unit (PIRU), Department of Health Services Research and Policy London School of Hygiene & Tropical Medicine London UK

**Keywords:** framing, public attitudes, shared funding, social care, social care funding

## Abstract

Twelve synchronous online focus groups were conducted, each involving four to six members of the general public who had expressed in‐principle support for sharing the costs of social care for older people between service users and government. These explored participants' reasons for preferring a shared approach and their views on how costs should be shared, with particular attention given to the sociocultural frames employed. Four main sociocultural frames were identified, reflecting dominant discourses concerning (i) the financial burden of meeting social care need (‘scarcity' frame) (ii) the core purpose of social care (‘medicalised conception of care' frame) (iii) the role and perceived limitations of the private market (‘consumer' frame), and (iv) fundamental concerns about safety, security and belonging (‘loss and abandonment' frame). Of these four frames, the ‘scarcity’ frame was dominant, with views about how costs should be shared overwhelmingly formulated upon assumptions of insufficient resources. This was reflected in concerns about affordability and the consequent need for the financial burden to be shared between individuals and government, and resulted in a residual vision for care and anxieties about care quality, cliff‐edge costs and abandonment. The concept of shared funding was also employed rhetorically to suggest an equitable approach to managing financial burden, reflected in phrases such as ‘*splitting the difference*’. Whilst out‐of‐pocket payments were sometimes seen as useful or necessary in the context of scarce public resources, the idea of shared funding was sometimes interpreted more flexibly to include individual contributions made in a range of ways, including tax, social insurance payments and wider social and economic contributions to society. Despite the dominance of the 'scarcity' frame, participants favoured greater government contribution than currently. These four frames and their associated discourses provide insight into how the public ‘hear’ and make sense of the debate about social care funding and, specifically, how apparent support for shared public–private funding is structured. Government and those hoping to influence the future of social care funding need to promote a vision of funding reform and win support for it by actively engaging with the sociocultural frames that the public recognise and engage with, with all of their apparent inconsistencies and contradictions.

**What is known about this topic**

1. Shared public–private funding options have featured strongly in debates on social care funding reform.
2. Public understanding of the social care system and how it is funded is known to be poor.
3. Whilst much previous research has shown that the public want social care to be funded on the same footing as the NHS, there is evidence of increasing support for shared funding.

**What this paper adds?**

1. Those expressing in‐principle support for shared funding employed four main sociocultural frames; a ‘scarcity' frame, a ‘medicalised conception of care' frame, a ‘consumer' frame and a ‘loss and abandonment' frame.
2. The ‘scarcity' frame was dominant, giving rise to a residual vision of social care and anxieties about care quality, cliff‐edge costs and abandonment.
3. Out‐of‐pocket payments were considered useful or necessary in the context of scarce public resources. The concept of shared funding was also used rhetorically, reflected in phrases such as ‘splitting the difference’. However, the idea of individual contribution was often interpreted flexibly to include collective forms of contribution, and participants favoured greater government contribution than currently.

## 1 INTRODUCTION

The system for funding and providing social care in England is widely perceived as unfair. Currently, those with £23,250 or more (upper capital limit) in savings and assets are ineligible for publicly funded care. This includes, unless a dependent or spouse lives there, the value of a privately‐owned home. Only those with £14,250 or less (lower capital limit) are entitled to fully publicly funded care, with savings and assets of between £14,250 and £23,250 considered on a sliding scale. Those eligible for publicly‐funded care also contribute all of their income to pay for their care keeping only a personal allowance (£24.90 per week for residential care, £189 per week for community‐based care) for living expenses. Reforms due to be implemented in October 2023 introduce an £86,000 lifetime cap on the costs of personal care (subject to various limitations) and an increase in the lower and upper capital limits to £20,000 and £100,000, respectively. These changes are to be funded through a new Health and Social Care Levy (HM Government, 2021). Most countries are similar to England in providing more comprehensive coverage of healthcare than social care needs, however the gap in public funding between health and social care is generally greater in England (Robertson et al., 2014).

As a result of stringent means testing, people needing care in England can find themselves subject to unpredictable and potentially catastrophic costs, with social care provision marked by market failure; a lack of insurance options, a fragile provider market, unmet need; inconsistent care availability and quality as well as poor workforce pay and conditions (Warren & Bottery, 2021). Despite more than twenty years of policy debate in England, involving at least five independent reviews and 12 Green and White Papers, reform of the social care system has been slow and incomplete (Foster, 2021; Jarrett, 2017; Thorlby et al., 2018). Within this debate, shared public–private funding options have featured strongly. These have included a funding cap (Dilnot, 2011), which came close to being implemented during the Coalition Government (2010–2015) and is in the process of being adopted, in modified form, by the current Government (2019‐present). Other proposed funding reforms involving shared public–private funding have included social insurance models, such as those in Germany, the Netherlands and Japan (Hemmings & Curry, 2020; Schlepper, 2021).

Internationally, social care funding arrangements and their associated historical and cultural contexts vary considerably, with public attitudes about social care provision heavily context dependent (Cylus et al., 2018; Fernandez & Forder, 2010; Sussex et al., 2019). However, where significant reforms have taken place, as they have, in the Netherlands, the United States, France, Germany, Japan, Korea and Ireland, for example, these have taken time, often involved successive failed attempts and required the development of high levels of public and political consensus (Robertson et al., 2014). In England, we know that public understanding of the social care system and how it is funded is poor (Bottery, 2019; Ipsos MORI, 2018), with consequences for the level and quality of public debate concerning potential reforms. For example, reform attempts under the Brown (2007–2010) and May (2016–2019) Governments were misleadingly but successfully dubbed, respectively, a ‘death tax’ and ‘dementia tax’ by political opponents.

Research has commonly found that the public want social care provided on a similar footing to the NHS or, sometimes, mistakenly believe this is already the case (Bottery, 2019; Ipsos MORI, 2018; Sussex et al., 2019). However, there is evidence of growing support for sharing costs between individuals and government (Gregory, 2014; Overton & Fox O'Mahony, 2016). Longitudinal data from the British Social Attitudes (BSA) Survey and NatCen Panel Survey, for example, have recently found that support for individuals paying up to a cap and government paying the rest has overtaken support for government‐only funding for the first time since 2012 (Curtice et al., 2022). Similarly, a recent national survey of public attitudes undertaken by some of this paper's authors found that as many as 58 % of individuals thought older people's care costs should be shared, with the state paying a larger share of costs than currently (Read, Erens, et al., 2021; Wittenberg et al., 2022). Whilst evidence, therefore, suggests that public attitudes to social care funding in England are evolving towards greater acceptance of shared forms of funding, we still know little about what people have in mind when they express support for shared funding in such surveys.

In this study, we aimed to identify how people who express in‐principle support for shared funding for older people's social care understand and explain their preferences, with special reference to the sociocultural frames they employ.

## 2 METHODS

### 2.1 Conceptual framework

We adopted a framing approach (Goffman, 1974). Schön and Rein (1994) note that policy debates can become unproductive where participants view the issues through different ‘frames’. Identifying these can help clarify underlying value frameworks, focus debate and shape more effective communications. The framing approach differs from a classical rational approach to policy preferences, which assumes people have access to good information, time and capacity to comprehend it and the ability to identify and meaningfully compare different options. We know these conditions do not hold in the case of social care funding; the public find the topic highly complex and have limited, sometimes flawed, knowledge of how it works, or could work. In such circumstances, culturally shared ‘frames’ (e.g. in the form of simplified narratives, myths, analogies or generalisations) provide simplifying structures that allow people to make sense of complex and ambiguous information (Elwell‐Sutton et al., 2019; Frameworks Institute, 2018). Frames have their own internal logic and, by emphasising certain aspects over others, encourage particular understandings of social problems. Rhetorically, framing can help create a sense of coherence, persuasiveness and obviousness (Rein & Schön, 1996). Frames may be promoted intentionally by those seeking to influence public opinion or may reflect prevailing social and cultural perspectives (Rein & Schön, 1996). Frames are socially constructed and re‐constructed through social discourses involving media, politicians, prominent spokespeople and social networks (Diehl & McFarland, 2010; Goffman, 1974; Ross, 2000). To date, there has been only limited application of this approach to the issue of social care reform (Crowther, 2019, 2020; Social Care Futures, 2021).

### 2.2 Sampling, recruitment and data collection

**FIGURE 1:**
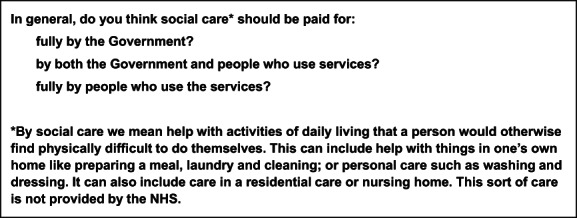
Screening question for recruitment.

We conducted 12 synchronous online focus groups between October 2020 and March 2021. Focus groups were selected for their effectiveness in exploring shared social meanings and generating rich data through participant interaction. To optimise effectiveness, we opted for slightly smaller groups (four to six participants) than would be usual face to face. We worked with the market research agency, Ipsos MORI, to recruit participants in England from the general population. Potential participants were asked a screening question and we invited only those expressing support for shared public–private funding (Figure 1). Groups were stratified by age (18–44; 45+) and we sought range and diversity by social grade, geography and ethnicity (Table 1). Three groups were reserved exclusively for minority ethnic participants to ensure adequate representation for analysis.

**TABLE 1:** Sample composition

Characteristics	Number participants, *N* = 58
Age	
20–29	8
30–39	14
40–49	18
50–59	15
60–65	3
Gender	
Female	29
Male	29
SEG	
B	25
C1	20
C2	12
D	1
Ethnicity	
Asian	9
Black African	9
Black Caribbean	7
Multiple ethnic backgrounds	2
Other	1
White British	30
Work status	
Currently not in paid employment	1
Full‐time education/studying	2
Full‐time employment	45
Look after the home/children	3
Part‐time employment	4
Retired	3
Housing tenure	
Live rent free	5
Own outright	7
Own with a mortgage/loan	34
Rent (housing association)	3
Rent (private landlord)	9
Region	
South West	23
South East	11
London	12
North	12
Urban/rural	
Urban	28
Rural	30

Groups were conducted online because of COVID‐19 pandemic restrictions. We used the free online video conferencing software, Zoom, which we believed most participants would have access to and be familiar with, although we also provided instructions and offered one‐to‐one support to set up the application. Groups took place at different times during late afternoon and evening. They were facilitated by the lead author with assistance from the second author and guided by a topic guide. We began each group by providing a brief explanation of the English social care system, including its funding, to limit factual disagreements and questions of clarification during discussions. The topic guide included general questions covering how costs should be split between government and individuals, covering both residential and home care and exploring participants' rationales for their choices (see supporting information). We also employed vignettes to explore how participants' general views applied, or were rethought, in the context of specific individual cases. All participants were actively encouraged to contribute and engage informally in the shared discussions. Whilst it is possible that participant interactions were less generative than may have been the case in person, group discussions were generally lively and productive. Discussions were audio recorded with permission. Each participant received a £50 thank you for taking part.

### 2.3 Data analysis

Audio recordings were transcribed verbatim and analysed thematically by the first two authors using NVivo software (Koon et al., 2016). We followed Braun and Clarke's (2006) six steps for conducting thematic analysis: familiarisation, coding, generating themes, reviewing themes, defining and naming themes, and writing up. Specifically, transcripts were initially read for familiarisation and annotated. Data were then descriptively coded in NVivo, covering different reasons for supporting shared funding, respective benefits of public and private contributions and various contextual factors. Coding was initially undertaken at a high level of granularity. Through an iterative process, involving the grouping and re‐grouping of initial descriptive codes, informed by in‐depth discussion between the lead and second authors and consultation with the wider team, data were eventually organised into four main themes, each aligned with a separate frame and its associated implications.

Ethical approval was provided by the London School of Hygiene and Tropical Medicine Research Ethics Committee, Reference 21,783.

## 3 FINDINGS

We identified four main sociocultural frames employed in discussions about shared funding for social care for older people:

- scarcity
- medicalised conception of care
- consumer
- loss and abandonment.

The ‘scarcity' frame was dominant, with the three other frames premised upon it. The frames were occasionally challenged but challenges were generally isolated and not developed by other participants. Participants tended to draw upon the frames flexibly and multiply, sometimes in exploratory ways, and struggled to sustain a single normative standpoint.

I've wavered as the conversation changes. You think a bit more broadly about the whole situation, so your initial thoughts can change.

I mean it's so difficult. It's so difficult that, like, I've changed my mind four or five times now.

Whilst groups were designed to reflect diversity, we found limited differences by age, ethnicity or other participant characteristics. Below, we describe the four frames and associated views on shared public–private funding for older people's social care.

### 3.1 Scarcity, difficult choices, sharing burden and finding a ‘fair split’

Discussions were dominated by a ‘scarcity' frame. Neither government nor individuals were thought able to afford social care alone. Participants described mounting demographic pressures and competing governmental priorities, including health, education and COVID‐related commitments and debts.

The government cannot pay for everything because they are running out of money. Well, they have run out of money, end of story.

For some, the sustainability of the social care system depended upon individuals paying higher out‐of‐pocket contributions.

It's at breaking point at the moment … the more responsibility we pick up as individuals could actually holistically help the whole social care system.

However, others thought individuals were paying high taxes already and may also have been affected financially by the COVID‐19 pandemic. These affordability challenges were often seen as potentially catastrophic.

The population's rising and if everybody finds themselves in need of social care, then, you know, it could spiral, couldn't it? And then, the government finds itself in a situation that they can't sustain, and that the individual can't sustain.

Lack of political leadership for resolving these challenges was also identified.

I guess if the government are not prepared to put the money in, or not prepared to ask us to put the money in, then nothing is happening.

As a consequence, existing, particularly publicly‐funded, social care was widely considered difficult to access and of poor quality, with few protections for those unable to afford care.

I'm guessing there's people right now, poorer people who are dying because they can't afford the care and they can't afford to go into care homes.

Occasionally, the idea of scarcity' was challenged, although these comments tended to be isolated and not expanded upon by other participants.

I do think there's money out there because the Government has actually raised billions and billions toward this COVID pandemic, so there's money out there to be used, so why can't they do it for the care sector after the COVID's all finished?

Scarcity framing was associated with a perceived need to limit government spending and make *‘difficult choices.’* These choices were commonly experienced as complex and ethically demanding.

Who do you assist? Do you assist person A or person B? There is no sort of great answer, but somebody has to pay the bill and that's the sad fact of it.

I find it really difficult. It's almost like saying ‘let that person die before that person because he's more important.’

In this challenging context, shared funding was understood primarily as a way of sharing financial burden. Participants often did not know exactly how costs should be shared between individuals and government but many opted for a fifty‐fifty split, at least as a starting point, on the basis of their ‘*gut feeling*’ or because it ‘*feels fair*.’ Others suggested ‘out‐of‐pocket’ contributions ranging between 25 and 75 %, depending on individual circumstances, or expressed uncertainty. However, sharing the costs of care was not just presented pragmatically, as a way of meeting financial challenges. The idea of sharing costs was also employed rhetorically to convey a sense of fairness and compromise, with a fifty‐fifty split seen as the epitome of reasonableness. This was expressed using phrases such as *‘splitting the difference’, ‘both ways*’, ‘*fair for both sides*’, ‘*split fairly*’, *‘a contribution both sides*’, ‘*a mutual thing’, ‘even across the board’, ‘fair across the board’, ‘equal across the board*’, ‘*a balance*’ and ‘*fair shares*’. .

Why should the government pay more or why should I, say if it was me or whoever, pay more? It's a mutual thing then. It's fifty‐fifty. No‐one can really argue the situation.

Notably, however, in discussions about specific individuals (involving vignettes), participants tended to favour a split involving a greater government contribution, often upwards of 75 %. A split of fifty‐fifty or with government paying the lesser proportion tended to only be proposed where an individual was perceived to have considerable wealth. Nonetheless, a fifty‐fifty or similar split was sometimes seen to improve on existing arrangements, which were thought unfairly weighted towards individual contribution.

I'm not entirely sure but half and half just seemed a place to start. I know on occasions I've been dealing in them sorts of things and it's one hundred and zero, and that did not seem right.

A fifty‐fifty, or similar, split was also thought more transparent than the way costs were currently split between individuals and the state and as ‘*easy to work with*’. It was also occasionally employed heuristically as a way of managing uncertainty and risk, including with regard to the level and duration of future care needs.

You cannot control how ill someone's going to get or you do not know what's going to happen in the future or how bad someone's going to get, so I would say fifty‐fifty.

Many participants were ambivalent about whether, and to what degree, housing assets should be used to pay for care. In the context of scarcity, some thought that those with greater housing wealth should pay more so as not to deprive others less able to pay. However, it was widely thought that no‐one should '*lose everything*'. As a way of balancing these competing considerations, participants commonly employed the same heuristic concepts of finding *‘a balance*’ or *‘a fair split*’.

### 3.2 Medicalised conception of care, prioritising bodily over non‐bodily care

Participants commonly prioritised social care that they perceived to be related to medical conditions for public funding, particularly if were to be recommended by doctors or other authorities.

If they are going in because they need to, and the families and they just cannot look after themselves anymore, then maybe fifty‐fifty, but if the hospital or the doctors have actually referred them to go into a care home, I do think then that's when government should step in a little bit more.

The idea that medically‐related needs could be meaningfully distinguished from broader social care needs was only occasionally challenged.

Someone who's frail and elderly is going to need ongoing help, although you might not class it as a medical issue.

At its extreme, this frame was understood as prioritising public funding for care focused narrowly on physical needs, urgent circumstances and, sometimes, survival.

Basic care should just be to keep you alive. I guess anything else on top of that's a luxury.

Their [out‐of‐pocket] contribution should be a lower amount because it's pretty much desperate palliative care

Commonly, it was thought that non‐bodily care such as shopping or preparing food, or assistance to leave one's home or participate in social, cultural and community activities, should be funded privately, or provided by families or charities.

Getting out to socialise, how strong a need is that? That the state should share in the burden of that cost, is debatable in my mind, in comparison with other more severe needs.

When discussing specific individuals (using vignettes), however, participants often came round to thinking that these wider types of support should attract some public funding but, to justify this, the underlying needs were sometimes redefined as health related. For example, it was suggested that *‘people die of loneliness more so than anything else,’* with loneliness described as *‘an epidemic*.’ Similarly, it was thought that socialising in dementia may need professional facilitation and therefore ‘*may be a health thing as well*.’

### 3.3 Consumerism, choice, disparities and limits of the consumer model

Social care was sometimes framed in consumer terms. With publicly‐funded care widely considered difficult to access and of poor quality, it was often thought that private funding could help to fund higher quality care, either by augmenting government funding or purchasing privately‐available services.

The different levels, there's like gold, silver, bronze, so if I can afford to go into a gold one with my assets then I'm going to do that. I might as well enjoy the fruits of my labour.

The ‘consumer' frame was used to justify the use of savings and assets to fund good quality care, with this seen as spending on, or indulging, oneself.

I think they should use as much as they need of their assets to be cared for because they have worked for it and they deserve to be looked after and cared for, and their assets should be spent on them.

Social care was sometimes compared to education, in which public and private systems were seen to run in parallel, as well as to commercial services such as hotels and funeral services. Exercising choice was often emphasised.

It's like anything in life, you have a choice and you have to pay for bettering it.

Framing social care as a consumer good also provided a framework for justifying disparities in the level and quality of services that people receive.

If you have saved up money and then been successful and done things in your life and you can afford better care then, yeah, that's fair game as well.

Viewed through this frame, participants sometimes found it difficult to accept that people should receive comparable care regardless of their financial contribution.

In a care home, there are people who come with zero balance. Those people will be still eating the same money, the same food. They're getting the same services as the person who is paying, which is not fair.

The consumer frame, however, was the most challenged of all four frames. For example, participants sometimes expressed concern about the inequities inherent in a consumer model of care.

When it comes to care and health, I think everybody should get good, when I say basic, I mean good care. It should not just be the elite.

Respondents also struggled to identify discretionary aspects of care that were suitable for provision via the market, occasionally resorting to hyperbolic examples.

Where you want a Jacuzzi in your room or a really [all laugh] big room then, yeah, you have to pay for it.

In residential care, nicer environments and hotel services were identified as extras one could pay for. Additional staff time, attention and responsiveness were also discussed.

The people that can afford the extra stuff, that's maybe like, you know, a buzzer that's 24 hours or something like that.

Other participants suggested that if public funding was prioritised for medically‐related needs, private funding could be used to enhance other aspects of care.

People are all on the same level, living the same type of standards, but you have much more activities [on gold], say, than you would on the bronze or silver.

Private provision was also seen as potentially increasing the overall level of resources available, thereby helping to sustain a fragile social care system.

### 3.4 Loss and abandonment, solidarity, security and personal responsibility

Participants emphasised the personal and emotional impacts of existing social care funding arrangements. Prominent amongst these were feelings of loss and abandonment.

We're all going to get old, it's going to happen to all of us, so we do have that accountability and we do need to think about it, but at the same time, it does not mean that, once we are no longer of use, and we are not earning any more, we should just be abandoned by the government.

Participants envisaged or described feelings of shock and distress at *‘cliff‐edge*’ costs and anguish about the sudden, unplanned loss of housing assets, built up over a lifetime, to meet these. The depletion of assets over a short period of time to pay for care was often understood as having assets *‘taken away*’ or individuals being ‘*penalised*’, especially if care received was considered *‘basic*’ or poor quality. This contrasted with the ‘consumer frame’, where savings and assets could be used to purchase better quality care and additional comforts.

One of the frustrations with losing your assets is it hangs like a chain round your neck for so many years. You try to work to pay off your mortgage, and then for someone to just take it away, just so you can live such a basic lifestyle, feels unfair.

Participants were also concerned about being abandoned when their money ran out.

That money goes really fast, really super‐duper fast. So what's going to happen if it goes, are you just going to be kicked out, and, what happens then?

It was noted that those affected would already be experiencing significant and challenging losses, of health and independence, and perhaps approaching end of life. Participants also worried about families; whether spouses would be turned out of their homes to pay for care and about the difficulties they could face coming to terms with the loss of an expected inheritance, one their relative had wanted for them, whilst simultaneously coping, potentially, with grief, loss and providing informal care.

He lived to be 97, and you just think all that he's worked for, it's all gone, and he was upset before he really went downhill, because everything had gone, everything he wanted to pass on had gone.

The ‘loss and abandonment' frame was associated with preferences for greater levels of tax‐based funding, risk pooling and collective provision, with comparisons made to funding the NHS.

You'll get people that might not ever need it, but then that's the same for hospital isn't it, for anything that we pay for. We might never need it. It's an insurance policy.

Despite an emphasis on risk pooling and collective provision, the importance of *‘personal responsibility*’ or *‘personal accountability*’ was commonly stressed. In practice, however, these concepts were closer to ideas of reciprocity and citizenship, involving the idea of making a commitment which could be demonstrated through various forms of contribution including tax and social insurance payments, as well as, potentially, out‐of‐pocket payments. Indeed, the type and level of contribution was also often considered less important than its social and symbolic value.

I think it's important that if the individual can contribute it shows their level of commitment as it were.

In fact, participants commonly thought out‐of‐pocket payments were more difficult to plan for and could be experienced as a shock, and so preferred to put money away over time. They sometimes used the example of pension savings, potentially involving an employer contribution, with the resultant savings pot purchasing something comparable to a pension annuity so that care costs could be met for as long as needed without fear of money running out. Alternatively, participants wondered whether they could purchase private insurance.

We should be funding our own care by way of a pension, or an insurance policy. It should not be left to dwindling savings, when you are no longer working.

However, participants sometimes thought that out‐of‐pocket payments could encourage more saving, incentivise people to take more care of their health and discourage unnecessary or excessive demands on the system.

It also acts as a hindrance for people who may not necessarily need the care. If they know it's completely free, they could totally abuse it, but if they know they have to pay, say, 25%, they might think twice.

Some felt that people are owed social care in recognition of social and economic contributions made during their lifetime. Participants, however, worried that younger people might be unwilling to pay for older people's social care if not assured of similar support in the future (although no younger participant expressed such views). Less commonly, participants argued that people should receive support solely because they are human and experiencing vulnerability. This was sometimes conceived of in terms of universal entitlements and sometimes as something owed specifically to those in financial need.

I think people should care, as a matter of being alive, care for each other.

You have people who have nothing and are perhaps on their own and isolated and have very little resources at all, and hopefully as a Western caring society, we need to look after people in our society.

## 4 DISCUSSION

Our study identified four main sociocultural frames. These reflected dominant discourses concerning, respectively, the financial burden of meeting social care need (‘scarcity' frame), the core purpose of social care (‘medicalised conception of care' frame), the role and perceived limitations of the private market (‘consumer' frame) and fundamental concerns about safety, security and belonging (‘loss and abandonment' frame). These frames and their associated discourses provide insight into how the public ‘hear’ and make sense of the debate about social care funding and, specifically, how support for shared public–private funding is structured and understood.

The ‘scarcity' frame was dominant, holding that there are few resources available to pay for care, that neither government nor individuals can afford to pay for care alone and that the financial burden needs, therefore, to be shared. Scarcity also necessitates *‘hard choices*’ about allocating public funds, which are potentially ethically challenging. This framing reflects over a decade of policy‐making in England involving significant public spending cuts, as well as media narratives about financial crises in the sector, and poor and abusive care (Bottery, 2020; Crowther, 2020; Mulley, 2011; Read, Wittenberg, & Mays, 2021).

The three remaining frames are predicated upon the ‘scarcity' frame. A ‘medicalised conception of care' frame prioritises public funds for care perceived as medically necessary. The idea of medically necessary, however, was ill defined; some suggested referral by doctors, others defined it as bodily care that someone could not do for themselves because of a health condition. Non‐bodily and social aspects of care, in contrast, were commonly considered less essential and, in a context of scarcity, thought more appropriately provided privately, by family or by charities. However, when discussing specific examples (using vignettes), participants often came to consider non‐bodily and social needs as more important, but sometimes recast them in medical terms to justify public support. The ‘medicalised conception of care’ frame reflects the dominance of the Western biomedical model of health and disease (Monaghan & Bury, 2022), historically differentiated patterns of funding for health and social care and a distinction between health care, where professionals decide what care people need and receive, and social care, where individuals and families are perceived, whether accurately or not, as exercising greater discretion and choice, with this, in turn, associated with the idea of discretionary spending. The 'medicalised conception of care' frame is also associated with a highly residual vision of care, precluding more holistic understandings of what social care can achieve (Dean, 2020; Think Local Act Personal & Coalition for Collaborative Care, 2018; Warren & Bottery, 2021). F Crowther (2019, p.9), for example, defines social care as potentially helping us all to *‘live in the place we call home with the people and things that we love, in communities where we look out for one another, doing the things that matter to us'.?*.

A third frame considers social care from a consumer perspective. It was sometimes thought that, given the context of scarcity, privately‐funded care could help to address limitations in public provision. However, some perceived there to be little or no link between private funding and better quality care. In practice, participants could also identify few aspects of care for which market‐based variation in access and quality was considered acceptable; where it was, this was limited to hotel‐type services; staff time and responsiveness; and support to participate in social and cultural activities. It was widely thought, however, that access to good basic care should not depend on ability to pay.

The fourth frame focuses on experiences of loss and abandonment. This includes distress and anxiety associated with sudden ‘cliff‐edge’ care costs, money running out and concerns about care quality and access, all coming at a time when people are already experiencing vulnerability. It was thought that families could feel similarly abandoned, left to come to terms with loss of inheritance that their relative had wanted for them, whilst simultaneously coping with personal loss and concern for their relative. This frame is associated with a desire for more risk pooling and participants strongly emphasised ideas and concepts that resembled those of reciprocity or citizenship, although they tended to use individualistic language (e.g. personal responsibility) to express these. In this way, participants effectively built a rhetorical ‘bridge,’ helping to reduce dissonance between ideas of making an individual contribution, which they had expressed in‐principle support for, with support for greater risk pooling and collective provision (Benford & Snow, 2000).

Participants generally found it difficult to maintain a single normative position, commonly moving between different frames and sometimes expressing confusion and uncertainty (Srblin et al., 2015). They also tended to think about social care funding predominantly in terms of personal and emotional impacts.This sits uncomfortably with a public debate that has tended to focus on the costs of care rather than people's experiences and values.

A discourse of scarcity over more than a decade may well have led some to see individual out‐of‐pocket payments as unavoidable (Read, Wittenberg, & Mays, 2021). Shared funding was valued as a way of balancing the respective strengths and weaknesses of public and private funding; the perceived benefits of public funding included risk pooling, spreading costs and promoting a sense of belonging and inclusion, while the benefits of out‐of‐pocket payments were seen to be especially relevant in a context of scarce resources and included purchasing potentially better quality care and additional comforts, discouraging overuse of services, supporting a fragile public system and providing a fall back should governments be less willing or able to fund care in future. Notably, participants appeared unaware that private funders commonly pay more than local authorities for the same care in the same care homes. However, in the course of discussions, tax, social insurance payments and even social contribution were viewed as morally equivalent to making out‐of‐pocket payments, with these contributions understood in terms of the broader value of taking *‘personal responsibility*’.

Importantly, too, participants widely thought government should make a much larger contribution than is currently the case, commonly suggesting an average contribution of between 25 and 75 % and often proposing 75 % or more when discussing individual cases (vignettes). This was not always made explicit in discussions, perhaps because participants failed to appreciate just how far the current system relies on self‐funding. The concept of shared funding was also often understood more rhetorically, signifying fairness and compromise in the context of scarce resources. This was captured in myriad phrases such as *‘fair on both sides*,’ ‘*even across the board*,’ ‘*splitting the difference*’ and *‘fair shares*’. The concept was also employed heuristically as a means of parsing complexity or uncertainty. In practice, then, whilst accepting a rhetoric of *‘both sides*’ needing to contribute and the potential practical necessity of some level of out‐of‐pocket payments, many participants expressing support for shared funding appear to be still fundamentally committed to a predominantly public and collective system of funding for social care.

In their work on social movements, Benford and Snow (2000) describe different communication strategies, covering processes of bridging, amplification, extension and transformation. These strategies can support political actors and opinion formers to mobilise and countermobilise sociocultural frames and their associated discourses to influence public attitudes. Government and other stakeholders wishing to shape the future of social care funding need to promote a vision of funding reform and win support for it by drawing on these strategies and actively engaging with the sociocultural frames that the public currently hold and engage with, with all of their apparent inconsistencies and contradictions. It may also be of interest to conduct similar research to examine developments in public discourses about social care funding once the planned funding reforms in October 2023, which introduce changes to the upper and lower capital limits and the introduction of a lifetime funding cap, are fully implemented.

## 5 CONCLUSION

The views of those expressing in‐principle support for shared funding for older people's social care were overwhelmingly framed by an assumption of scarce resources, resulting in concerns about sharing financial burden, a residual vision for care and anxieties about cliff‐edge costs and abandonment. The concept of shared funding was employed flexibly and rhetorically by participants to attempt to address the multiple challenges that they perceived there to be in ensuring that scarce resources are able to stretch to meet people's needs. Our study provides a richer view of how the public interpret the debate about social care funding, and how they understand shared funding in particular, which policy makers and opinion formers should take into account when shaping funding options and seeking to win public support for proposed reforms.

## AUTHOR CONTRIBUTION

All authors contributed to the concept and design of the study. JD & JE conducted the focus groups and the analysis. JD drafted the manuscript. All authors reviewed and edited the manuscript and approved the final version.

## CONFLICT OF INTEREST

No conflicting interests.

## Supporting information


Appendix S1

## Data Availability

Research data are not shared.
