# Supplementary material for: The sociocultural framing of public attitudes to sharing the costs of social care for older people in England
Source: Health Soc Care Community. 2022 Aug 5;30(6):e5270–80. doi: 10.1111/hsc.13946 (PMC10087265; doi:10.1111/hsc.13946)
Supplement: Supplementary file 1 — Appendix S1 [file HSC-30-e5270-s001.docx]

Supporting Information

**Sharing the costs of social care: focus group topic guide**

**Total time: 1hr45mins**

# RESEARCHER INTRODUCTIONS/ HOUSEKEEPING/ CONFIDENTIALITY/ CONSENT (5 MINS)

- Introduce selves and PIRU (LSE and LSHTIM)
- Remind session will last 1hr 45mins
- Topic guide for coverage but informal discussion, more like a conversation. Invite to contribute freely, no right or wrong answers
- Remind participation voluntary, don’t have to answer questions if you don’t want to. Clarify we will not ask any personal questions but people can draw on own experiences if they wish to
- No-one will be identified in the report, any quotes anonymized
- Remind we will be recording the discussions; explain we wouldn’t be able to take notes fast enough and want to make sure that we don’t miss anything. Only the research team will listen to the recording and will be destroyed when the study is completed. Check consent.
- Any questions?

# GROUP INTRODUCTIONS (5-10 MINUTES)

Go around group and briefly (30 secs-1 minute each) give name and just one thing about us e.g. jobs we do/ did, children/ grandchildren, hobbies etc?

Also, if people wish to, very briefly, experiences of social care.

# SETTING THE SCENE (MAX. 15 MINUTES)

Start by giving brief information so all starting from the same place.

## What we mean by social care

By social care we mean help with day-to-day activities (known as activities of daily living) that people would be unable, or would struggle, to do for themselves. This includes

- - help provided to people in their own homes (known as home care or domiciliary care), and
  - help provided to people who are living in residential care homes/ nursing homes.

It can include, for example:

- - cleaning
  - shopping
  - laundry
  - preparing a meal

It can also include personal care, such as:

- - washing
  - dressing
  - help with getting in and out of bed or a chair
  - help with eating
  - moving around

This type of care is not provided by NHS.

People of any age may get social care, but we are mostly focusing on older people in today’s discussion.

## Demand for social care is rising

A growing and ageing population

- ‘baby boomers’ growing older
- living longer, with chronic illnesses
- Proportion of population 65+ years will increase from 18% to 25% in next 30 years
- Number of people 85+ will double (1.6 to 3.6 million) in next 30 years
- A quarter of people 65+ need help with ‘activities of daily living’

## How social care is currently paid for

- Most people who need social care pay for it themselves or get unpaid help from family or a friend
- Only get help if needs are serious and you have less that £23,250 in savings or assets
- Only get everything paid for if you have less than £14,250 in savings or assets
- If receive help, still have to contribute from income. Allowed to keep a personal allowance (£24.90 in residential care, £189 in community)

Notes:

- Serious needs: this is a high standard - it means you are not able to carry out most of your personal care or domestic tasks without help and support and that there will be a significant effect on your well-being
- Savings or assets: this means any money that is in saving accounts or the bank, stocks, shares or other savings. Your house is also counted among your assets if you (and your partner, if you have one) stop living in it (for example, to move into a care home)

Costs of care:

- Home care: approx. average £15 an hour.
- Care home: approx. average £700 per week (includes ‘hotel’ expenses).
- about 50% of people 65+ spend up to £20,000 in lifetimes
- about 10% of people 65+ spend over £100,000 in lifetimes

## Aim of today’s focus group

Different views about how social care should be funded.

- Some think it should be funded fully by the Government at and that people who use services should not pay anything, like the NHS.
- Some people think it should be paid for privately ‘out of pocket’, so without Government contributing.
- Some people favour a mix of Government support and private ‘out of pocket’ payments (around 60% said this in a recent survey we undertook).

You said it should be a mix when we asked you. We are interested in finding out more about your opinions.

There is no single right answer to the right amounts people and Government should pay or what the best methods for sharing costs are. More interested in understanding what sorts of things are important to consider.

# SHARING COSTS OF CARE

- We will begin with broad questions and go on to discuss more specific situations
- We will begin talking about care provided to people at home, later talk about care provided in a care home

## Care provided to people in their own homes (35 minutes)

Think about support provided to people in their own homes (home care). This is for people who are unable, or would struggle, to do day-to-day tasks for themselves. It usually includes things like shopping, cleaning and cooking. It can also include personal care, such as help with bathing or dressing and helping people take medications.

- - On average, roughly what proportion of the total costs of home care services do you think people using services should pay ‘out of pocket’ and what proportion should Government pay?
  - Ask people to mark a point on a spectrum, between two points representing Government pays everything and person using services pays everything (use online visual tools).
  - If people are unsure or say it depends, ask them to place themselves where they think, on average, the proportion should be and tell them you will give them a chance to explain their choice, including exceptions and caveats, in a moment
  - Individually ask participants [starting with the person furthest down one end, then the person furthest down the other end, and working towards the middle] why they put themselves there and not somewhere else?
  - What do other people think about these reasons? [generate as much discussion as possible around each of the reasons/ rationales raised by participants]?
  - Having listened to everyone, does anyone want to change their position? Ask why.

The above will be an extended discussion with extensive probing and generation of discussion.

Generate further discussion by presenting a small number of specific cases and examples constructed using the ‘case matrix’ below (these will be plausible cases, but generated randomly and be different for each focus group). Probe issues that arise. We can change details to gain more depth of discussion on specific issues identified by participants as important (e.g. asking people, *“what if we changed this characteristic?”*).

## Care provided to people in a care home (35 minutes)

This exercise will be conducted in a similar way to the previous one, with extensive discussion on the reasons for participants’ choices.

We will actively facilitate and probe the discussion to explore how funding of care provided in a care home should be similar or differ to funding of care provided to people at home.

Figure S1: Case matrix

|  | **CHILDREN** | | | | **LIVING** | | | **WEALTH** | | | **WEALTH/ SPOUSE CHILDREN** | | | **NEEDS** | | **AGE** | |
| --- | --- | --- | --- | --- | --- | --- | --- | --- | --- | --- | --- | --- | --- | --- | --- | --- | --- |
|  | Adult near | Adult far | Grandchild | None | Alone | Partner | Family/  friends | Own house | Pension | Income | Other’s house | Other’s pension | Other’s income | Needs > | Needs < | 65+ | 85+ |
| **CHILDREN** | | | | | | | | | | | | | | | | | |
| Adult children live nearby |  |  |  |  |  |  |  |  |  |  |  |  |  |  |  |  |  |
| Adult children live far away |  |  |  |  |  |  |  |  |  |  |  |  |  |  |  |  |  |
| Grandchildren |  |  |  |  |  |  |  |  |  |  |  |  |  |  |  |  |  |
| None |  |  |  |  |  |  |  |  |  |  |  |  |  |  |  |  |  |
| **LIVING** | | | | | | | | | | | | | | | | | |
| Widowed/single live alone |  |  |  |  |  |  |  |  |  |  |  |  |  |  |  |  |  |
| Live with partner |  |  |  |  |  |  |  |  |  |  |  |  |  |  |  |  |  |
| Live with other family/ friends |  |  |  |  |  |  |  |  |  |  |  |  |  |  |  |  |  |
| **WEALTH** | | | | | | | | | | | | | | | | | |
| Own house |  |  |  |  |  |  |  |  |  |  |  |  |  |  |  |  |  |
| Private pension |  |  |  |  |  |  |  |  |  |  |  |  |  |  |  |  |  |
| Income |  |  |  |  |  |  |  |  |  |  |  |  |  |  |  |  |  |
| **WEALTH/ SPOUSE CHILDREN** | | | | | | | | | | | | | | | | | |
| Own house |  |  |  |  |  |  |  |  |  |  |  |  |  |  |  |  |  |
| Private pension |  |  |  |  |  |  |  |  |  |  |  |  |  |  |  |  |  |
| Income |  |  |  |  |  |  |  |  |  |  |  |  |  |  |  |  |  |
| **NEEDS** | | | | | | | | | | | | | | | | | |
| More severe |  |  |  |  |  |  |  |  |  |  |  |  |  |  |  |  |  |
| Less severe |  |  |  |  |  |  |  |  |  |  |  |  |  |  |  |  |  |
| **AGE** | | | | | | | | | | | | | | | | | |
| Over 65 |  |  |  |  |  |  |  |  |  |  |  |  |  |  |  |  |  |
| Over 85 |  |  |  |  |  |  |  |  |  |  |  |  |  |  |  |  |  |

# CLOSING (5 MINS)

- Ask people if there are any points that they wish to raise that have not been covered in our discussions
- Thank people for their contribution
